# Patient-Centered Approaches for Designing Destigmatizing Sexual Pain-Related Web-Based Platforms: Qualitative Study

**DOI:** 10.2196/53742

**Published:** 2024-03-15

**Authors:** Abdul-Fatawu Abdulai, Hasti Naghdali, Heather Noga, Paul J Yong

**Affiliations:** 1 School of Nursing University of British Columbia Vancouver, BC Canada; 2 Women's Health Research Institute Vancouver, BC Canada; 3 Department of Obstetrics and Gynaecology University of British Columbia Vancouver, BC Canada

**Keywords:** stigma, digital health, sexual pain, destigmatizing, end user patients

## Abstract

**Background:**

Sexual pain is a common but neglected disorder that affects approximately 3% to 18% of women and an unmeasured number of gender-diverse people worldwide. Despite its wide prevalence, many people feel reluctant to visit conventional health care services or disclose their symptoms due to the fear of stigmatization. To alleviate this stigma, various web-based interventions have been developed to complement and, in some cases, replace conventional sexual health interventions. However, the way these web-based interventions are developed could inadvertently reproduce, perpetuate, or exacerbate stigma among end user patients.

**Objective:**

The purpose of this study was to understand patients’ perspectives on how sexual pain–related web platforms can be designed to alleviate stigma or prevent the unintended effects of stigma among patients who use web-based interventions.

**Methods:**

Individual semistructured interviews were conducted among 16 participants with lived experiences of painful sex in a large urban city in Western Canada. Participants were recruited via social media platforms, newsletters, and a provincial health volunteer website. Using a sample sexual pain website to provide context, participants were interviewed about their experiences of stigma and how they think web platforms could be designed to address stigma. The interviews were conducted via Zoom (Zoom Technologies Inc) and analyzed using thematic analysis.

**Results:**

The findings revealed 4 overarching themes that represented participants’ perspectives on designing web platforms that may alleviate or prevent the unintended effects of stigma. These findings suggested the design of inclusive web platforms, having a nonprovocative and calming user interface, having features that facilitate connections among users and between users and providers, and displaying personal testimonials and experiences of sexual pain.

**Conclusions:**

This study highlighted patient-centered design approaches that could serve as a reference guide in developing web platforms that alleviate or prevent the unintended effects of stigma, particularly among nonheterosexual and gender-diverse people. While this study was conducted in the context of sexual pain, the results might also apply to web platforms on other potentially stigmatizing health-related disorders or conditions.

## Introduction

Sexual pain is a common but neglected problem, predominantly experienced by females, that is estimated to affect approximately 3% to 18% of women globally [1]. The disorder affects multiple aspects of one’s life including absenteeism from work, poor interpersonal relationships, and impaired social functioning with a profound impact on the quality of life [1]. Despite the negative effects of sexual pain, many women feel reluctant to visit service providers or disclose their symptoms to family and friends due to the fear of stigma [2]. Stigma is defined as an attribute and dynamic social process characterized by widespread social disapproval, blame, rejection, devaluation, and segregation [3]. The extent to which people with sexual pain are affected by stigma depends on the nature of the disorder and whether it is concealed or exposed. According to Tyler and Slater [4], people with invisible conditions or disorders are more likely to be affected by internalized stigma, while those with visible conditions or disorders may be predisposed to public stigma. Public stigma refers to the overt discriminatory practices that are directed at someone, while internalized stigma refers to the acceptance of negative attributes and a reduced sense of self-worth as a result of possessing a supposedly stigmatizing attribute [2]. While hidden disorders like sexual pain may not immediately provoke public stigma, people with such disorders may still be concerned about an impending negative reaction from the public or even stigma from present and future intimate partners if their pain status is eventually revealed [4]. The fear of disclosing a condition can be classified as anticipated stigma, and this is quite common among people with sexual health–related disorders [5]. Therefore, people with sexual pain may face both internalized and public stigma of various proportions at the personal, interpersonal, and societal levels. As stigma becomes more of a dynamic social process [3], the modalities of managing this issue are becoming increasingly complex and challenging, especially for people with hidden disorders like sexual pain.

In response to the negative impact of stigma on people with sexual pain, various web-based platforms have been developed to complement, and in some cases, replace conventional care services [2,6,7]. Web-based platforms on painful sex could range from educational resources, decision support systems, web-based social support platforms, and therapy-based platforms [8-12]. Even though web-based platforms are increasingly used as resources inquiring about and managing sexual pain [13,14], available evidence suggests that sexual health–related web-based platforms could inadvertently foment stigma among end users [15]. Other studies revealed that merely using a sexual health platform in public spaces could be interpreted as having a disorder and could lead to stigma in what is termed stigma by association [6]. Notably, people who use sexual health–related web platforms may likely link the appearance of certain images and content to an existing social stigma, thereby resulting in technology-mediated experiences of stigma [7]. Furthermore, the replacement of face-to-face conversations with web-based tools may further emphasize the feeling of discomfort or stigma associated with in-person interactions as it gives an indication that sexual pain is not something to be discussed but self-discovered using web-based tools.

The likelihood of web-based platforms resulting in technology-mediated experiences of stigma may be attributed to the general lack of attention to the issues of stigma when developing sexual health–related digital platforms [16]. Even though patients’ views and opinions are usually sought when developing sexual health–related digital technologies [17,18], their perspectives on how web-based platforms can be designed to alleviate stigma are usually not considered when developing such interventions. The apparent lack of attention to stigma may not only lead to a mismatch between people’s expectations and web-based platforms but could inadvertently reproduce and perpetuate stigma among patients who may find such platforms undesirable [7]. We attempted to address this gap in our prior studies by developing a set of destigmatizing design guidelines to provide a reference guide for developers of sexual health–related web-based platforms [16,19]. However, these guidelines were based on expert opinions and did not address the salient concerns of patients regarding stigma. With a disorder like sexual pain, patients often withhold sharing their diagnosis publicly, and we believe that many of the stigma-related considerations for designing web-based platforms could only be obtained from people with lived experiences of the disorder. The purpose of this research was thus to examine the perspectives of people with sexual pain on how web platforms could be designed to alleviate stigma or prevent digital platforms from inadvertently fomenting stigma among end user patients. The findings of this study are expected to produce user-interface design recommendations not just for sexual pain digital platforms but for web-based platforms on other stigmatized conditions or disorders in sexual health and mental health.

## Methods

### Study Design

A semistructured interview was conducted among 16 people with lived experiences of sexual pain in a large urban city in Western Canada. Participants were recruited via (1) social media platforms, (2) newsletters, and (3) a provincial health volunteer research website. To be eligible, potential participants must have been at least 18 years old, experienced or self-reported sexual pain (alone or partnered), and reported previous or current use of a health-related web-based platform. Due to the limited to no involvement of male partners in prior sexual health–related studies [8], we decided to recruit people who also identify as biological males and report experiences of painful sex.

### Interview Guide

A semistructured interview guide (Multimedia Appendix 1) was developed based on the person-centered care model by focusing on the participants’ emotional needs and preferences about how digital health technologies can be developed to be less stigmatizing and emotionally safe. The interview guide was developed to address key areas including demographic information, participants’ experiences with stigma, and their perspectives on how web-based platforms can be designed to alleviate stigma or prevent the unintended effects of stigma. The interview guide was reviewed by our patient advisory team, pretested on 3 patients outside of this study sample, and was revised accordingly.

### Data Collection

The interviews were conducted by the first author (AFA), with assistance from the second author (HN). Since different web-based platforms exist, we decided to contextualize our study by providing participants with a sample website that we developed in a previous study [10]. A link to this website was sent to participants at least 3 days before the interviews. Participants, particularly those who have never used a sexual pain web platform, were asked to explore the sample website before the interviews. The interviews were conducted via Zoom with participants’ videos turned off to protect their confidentiality. Participants were asked questions related to the design of web-based content, interactive features, images, colors, sex, gender, and class representations on digital platforms that can help alleviate the stigma of painful sex. Depending on participants’ backgrounds and prior experiences of stigma, probing questions were asked regarding how web platforms can be designed to address the experiences of stigma related to their identity. Data collection occurred from April to July 2023, and each interview lasted approximately 1 hour. All interviews were recorded onto the University of British Columbia’s accredited Zoom Cloud and transcribed using TEMI web-based transcription services (Temi Inc).

### Data Analysis

The transcribed data were imported into the NVivo software (version 11; QSR International). Data were analyzed using a thematic analysis approach [20]. The data analysis occurred alongside data collection. The first and second authors familiarized themselves by reading over the first 4 transcripts multiple times. The 2 authors independently developed a coding framework inductively from the first 4 transcripts. We compared the 2 coding frameworks and arrived at a common coding scheme at the end of the fourth interview. Any differences between the coders were resolved by discussing with other members of the research team. We then applied the unified coding scheme to the rest of the data as they emerged from subsequent interviews. As the data analysis proceeded, the coding scheme was revised as new data emerged. The coded data were sorted and organized into categories, and themes were then generated from the categories depending on their overall importance to the research questions. We compared data from people in different age groups, sex, and gender identities to understand if such identities reflected specific preferences on how web platforms can be designed to alleviate stigma.

### Ethical Considerations

The study was approved by the University of British Columbia behavioral research ethics board (REB # H23-00273). All participants provided written informed consent before the data collection. All data were anonymous because participants were informed not to state or mention any identifying information during the interview. Each participant was provided with CAD $50, which was equivalent to US $35 honoraria in appreciation of the time spent on the study.

## Results

### Demographic Characteristics

A total of 16 participants were interviewed. Data saturation occurred after the 14th participant, and we decided to interview an additional 2 participants. Regarding gender identity, 12 participants identified as women, 2 identified as men, and 2 identified as two-spirit. Regarding racial identity, 8 participants identified as White, 3 identified as a person of color, and 2 identified as Indigenous. Participants’ ages ranged from 25 to 63 years, with the majority of them younger than 30 years old. Even though all participants had used health-related websites, 6 of them had additional experiences using sexual pain–related websites. While we did not specifically ask people about their sexual identity, 3 of them openly stated their sexual identity as nonheterosexual. Seven participants stated they are marginalized by being identified as a woman, nonheterosexual, and Indigenous. On one hand, participants who indicated a marginalized identity also reported feeling stigmatized at some point in their lives or a feeling of impending stigma from their experiences of sexual pain. On the other hand, participants who did not express any marginalized identities were not very concerned about stigma, with 1 participant indicating that they do not associate sexual pain with stigma.

### Participants’ Perspective on Design Approaches to Alleviate Stigma

The analysis generated 4 main overarching themes that reflected participants’ perspectives on how sexual pain–related web platforms can be designed to alleviate stigma or prevent such platforms from inadvertently fomenting stigma. Some of the broader thematic areas contain subthemes that come together to define each overarching theme.

### Theme 1: Inclusive Design of User Interfaces

#### Overview

Inclusive design emerged as a major theme that reflected participants’ perspectives on how digital platforms could be developed to prevent the unintended effects of stigma. Participants recognized the diversity of people who may have direct or indirect experiences of sexual pain and the need to design digital platforms that satisfy the experiences of people with different racial or cultural backgrounds and sexual and gender identities. Three subthemes emerged under the overarching theme of inclusive design.

#### Subtheme 1: Design to Include Diverse Sexual Experiences

The participants indicated that web platforms on sexual pain should not just be limited to information on penis-vagina sex but include other forms of sexual experiences. According to the participants, other forms of sexual experiences like anal sex and the use of sex toys could equally elicit pain and therefore must be captured on web platforms. Participant 3 stated saying:

Sex is not just penis to vagina interaction. There's also sex toys and different ways in which people partake in sex. And so being very inclusive of that sense so that everyone feels safe in that website environment. And touching on the fact that there are different toys and people who are trans, for example, use dilators which can also bring pain.

The participants stated that these other forms of sexual experiences are already stigmatized by society and excluding them from digital platforms could worsen the stigma associated with such sexual experiences. The desire for inclusion of information on diverse sexual experiences was common among younger people. In fact, 2 participants who were older than 60 years were not open to the idea of other sexual experiences beyond penis-vagina sex.

#### Subtheme 2: Design to Include Diverse Sexual and Gender Identities

The participants also recognized that people may have different gender identities apart from being a woman and still experience painful sex. They specifically stated that not all people who identify as a woman may have a vagina and not all people that identify as a man may have a penis. To accommodate gender-diverse people in web-based platforms, participants suggested having inclusive pronouns by having drop-down menus or subsections on websites that account for people with different gender identities. Other participants argued that including pronouns of all gender-diverse people on web platforms may be difficult or impossible to achieve. Thus, they suggested the use of gender-neutral terms as a way of reducing the stigma associated with other stigmatized sexual identities. Participant 8 stated the need for gender-neutral terms by stating:

Oh my god, my ovaries are partially removed because I had a tumor. And I think that using less gendered language would be very, very, very, destigmatizing for people like me and for people who are trans. Because I love when people actually think of us and it shows that we are not as bad as some people think. I would probably stick with non-gendered wording, like people with uteruses or just like talking about the specific parts that may show pains.

While participants argued for designs to include diverse gender identities, they agreed that painful sex may be more common among cisgendered people. In addition to the gender-diverse pronouns, participants suggested that web platforms on painful sex should include male partners who may not directly experience pain but may be part of the activity that produces sexual pain. Participant 6 noted the need to include male partners by stating:

But honing in on the partners aspect, I think is really to make sure that there are resources for partners as well who aren't actively dealing with the pain. Okay. It's something that I've noticed is missing a lot.

#### Subtheme 3: Design to Reflect Cultural, Religious, and Ethnic Diversities

The participants also indicated their preference for designs that include people with different cultural, religious, and ethnic identities. This theme occurred across participants who identified as Indigenous or other marginalized identities. Participants expressed the need to see their culture or ethnicity represented when visiting web-based platforms regarding painful sex. Participant 14 specifically stated:

I was talking to one of my friends and she's a black woman and she said when she was looking up sexual pain, she only saw white women on the websites. And so, she didn't think the information applied to her. She didn't see anyone who looked like herself. So, I think if images are used, it has to be intersectional so people can see themselves on the website.

According to participants, these minority groups are already marginalized and stigmatized in society, and such stigma may only intensify if their cultural and racial identities are not represented on web platforms. Some participants with religious backgrounds also noted the importance of religious sensitivity and how that can alleviate stigma, particularly stigma relating to traumatic sexual experiences. Participant 7 stated:

I think that having that cultural sensitivity aspect embodied in a digital platform is important. Whether that be having individuals who have religious trauma associated with sex and who have gone through this experience give a testimonial or talk about it on a video or having religious supports or access to resources on the website, whether it be clickable links, phone numbers, whatever it might be on there.

### Theme 2: Nonprovocative and Calming User Interfaces

#### Overview

Participants also reiterated the use of nonprovocative and what they termed “calming user interfaces” as an approach to alleviating stigma via web interfaces. According to participants, people with experiences of painful sex may already be overwhelmed by some form of hidden trauma and stigmatized feelings associated with the disorder. Thus, they suggested that calming and uncluttered interfaces could be an appropriate design approach to alleviating such stigmatized feelings. They specifically argued for the content on the landing pages to provide comfort to website users before they dive into the actual content of the website. The use of (1) nonprovocative and nonexplicit images and (2) subtle and calming colors emerged as subthemes that together defined the overarching theme of uncluttered and calming user interfaces.

#### Subtheme 1: Nonprovocative and Nonexplicit Images

Many participants argued for the use of nonexplicit images. Participants with a health science background thought explicitly displaying anatomical images of sexual organs might help in educating patients on the specific location of pain. Participant 2 stated:

For me personally, I feel like the more graphic and anatomical images, the better because when I was younger, I literally didn't know anything about sexual anatomy. it's probably not allowed on the internet, but I think having those images would be helpful.

However, the majority thought that such images could be uncomfortable and might inadvertently foment stigmatized feelings. Participant 1 stated her abhorrence for seeing explicit content on sexual health–related web platforms and indicated how that made her feel uncomfortable.

I read on one medium where they have provocative pictures of people, you know, in the lovemaking act. It wasn't pornographic but certainly explicit and personally, I am uncomfortable with that.

#### Subtheme 2: Subtle and Calming Colors

Participants’ views on addressing stigma via web platforms extended beyond the use of nonexplicit images to include the color of the images and the general color scheme of websites. There was unanimity in participants’ preference for colors like blue, green, dark, purple, soft greens, turquoise, and so forth. These colors were perceived to be calming and comforting for an already distressed user population of people with painful sex experiences. On the contrary, participants were generally unfavorable to bright colors like red and pink as such colors were thought to symbolize pain. Participant 9 indicated his desire for calming colors by stating:

so probably not like bright pink and that kind of thing. You know painful sex comes with its own trauma and having all these bright colors may be a put-off. I liked the one I saw in this interview, it was like really calm colors, and they weren't too noisy, so you really focused on the information on the site and it was like, I think calming colors are maybe a good idea too.

Apart from indicating how these calming colors may be comforting to the eyes, participants were not explicit on how these color schemes could help address stigma when probed further.

### Theme 3: Interpersonal Connection via Web Platforms

#### Overview

As a way of addressing stigma, participants indicated a preference for integrating interactive features on web-based platforms that allow the creation of interpersonal connections among people with similar experiences or with health care professionals. According to participants, being connected to others will be crucial in addressing stigma for a hidden disorder like sexual pain. Two subthemes emerged under this overarching theme.

#### Subtheme 1: Creating Forums to Connect People With Similar Pain Experiences

There was a strong preference for forums, discussion boards, and messaging boards that would enable people with similar sexual disorders to connect, interact, and share experiences with each other while remaining anonymous. Participant 9 compared this to Reddit, and how a Reddit-like function on web platforms can offer a free, easy, and anonymous forum for people to discuss sensitive topics.

I think in that sense; the forums open up the isolation. The experiences and stories of strangers who have the same condition and you know such varied experiences will tell me that I am not alone in this and should not harbor any negative feelings. You know the internet gives us this fantastic gift of connection without obligation.

While participants acknowledged the importance of forum posts in addressing stigma, they also recognized the security and privacy risks associated with forum posts and how they can inadvertently perpetuate stigma. One participant noted how such forums can be used as avenues for harassment, bullying, and other risky behaviors. To prevent digital health platforms from inadvertently fomenting stigma, the participants suggested developing rules and regulations that govern forum posts, the use of pseudonyms and having designated professionals who would moderate web-based platforms.

#### Subtheme 2: Creating Avenues to Connect With Health Care Professionals

In addition to creating avenues on web platforms for people to connect, participants also reiterated the importance of creating avenues to connect with health care professionals, especially if it is a local website. According to participants, connecting with health care professionals via web platforms can “serve as a shield” from being exposed to the public stigma—a situation that participants found helpful during the COVID-19 pandemic. Participant 13 noted the importance of connecting to professionals by stating that:

speaking to a medical professional is more easily accessible and then having kind of the barrier of the screen in between, like not having to talk to someone face to face about it, but be able to type, I think could be helpful and, and perhaps start as a way to reduce the stigma.

According to participants, these forms of web-based interactions via web platforms can facilitate web-based consultations, thereby reducing the stigma people encounter by visiting onsite services.

### Theme 4: Displaying Personal Accounts and Experiences of Painful Sex

The data revealed that displaying personal experiences on web platforms could help alleviate the stigma of painful sex among web users. The participants stated that seeing or hearing what others have gone through can help them relate to other people’s personal accounts of sexual pain while validating their own experiences. Participant 2 stated:

When you find websites like this sharing information, it makes you feel like, I'm really not that strange. I'm not weird. Like there are a lot of other people who experience this too and that's why they're putting forward this information on this platform. So, it kind of makes people realize that it's more common than they think. And that also helps to alleviate fear surrounding it.

Some participants suggested the use of video testimonials to convey experiences, while others suggested a combination of text and video testimonials but from different sources. According to participants, reading about people’s stories; hearing their voices; and understanding what they went through, how they discovered their diagnosis, and how it was treated will help others learn and understand their situation and clarify myths and misconceptions that often fuel stigma. Some participants also suggested the use of comic images and videos to convey their experiences of painful sex as a way of addressing stigma. Participant 3 stated:

I like the comic thing. Like having people talking about these sorts of things in a comical style and that can make it less terrifying. By making it comical, you make it more entertaining, and people are more likely to pay attention to these sorts of things.

## Discussion

### Principal Findings

This study examined the perspectives of people with lived experiences of sexual pain on how web-based platforms can be designed to alleviate the unintended effects of stigma. While we acknowledge current practices of involving end users in developing sexual health–related digital interventions [21,22], no study has specifically investigated how such technologies can be designed to address the unintended effects of stigma. To the best of our knowledge, this is the first study that examined patients’ perspectives on how to address stigma through the design of digital health web platforms. Our study identified 4 broad categories that represented patient-centered strategies on how sexual health–related web platforms can be designed to address stigma. These categories include the design of inclusive web technologies, having a nonprovocative and calming user interface, having features that facilitate connections between users, and displaying personal accounts and experiences of sexual pain on web interfaces. Our sample revealed varying demographic information that ranged from marginalized populations including Indigenous, nonbinary, and people of color to White or Western populations. Participants’ indication of a marginalized identity suggests that the stigma of sexual pain may not exist in isolation but may intersect with multiple stigmatized identities to produce what is known as intersectional stigma [23].

These findings may help provide a conceptual or practical user-interface design guide for service providers to design digital health intervention that lessens or avoids the potential of fomenting stigma among people using sexual pain–related web platforms. We believe that these findings are not just applicable to sexual pain web platforms but could be applied to web-based platforms on other stigmatized conditions or disorders. By including male participants, this study also adds another dimension to sexual pain research in what is generally considered to be a woman’s health problem. The inclusion of people who identify as men male partners might have addressed the general lack of men’s involvement in sexual health research [24]. Even though participants who identified as men were not necessarily partners of those who identified as women as suggested in our prior study [10], they nevertheless illuminated the aspects of sexual pain that are specific to men yet often overlooked (eg, pain resulting from men who have sex with men).

### Strategies to Addressing Stigma in Web Design

The themes identified from this study reflect patients’ perspectives on how web-based platforms can be designed to alleviate the stigma of sexual pain. The findings highlight the importance of inclusive user interfaces for people with diverse sexual pain experiences; gender-diverse people; and people with different cultural, ethnic, and religious identities. The findings of this study thus extend the idea of inclusive design beyond addressing the usual age, physical, mental, language, and accessibility limitations [25-27] to include specific stigma-related concerns that are often associated with people of diverse sexual and gender identities (two-spirit, lesbian, gay, bisexual, transgender, queer, and questioning, intersex, asexual) as well as diverse sexual preferences (anal sex and sex toys).

It is worthwhile to note that sexual pain is often discussed in relation to heterosexual encounters, people who engage in anal sex or sex using vibrators or sex toys may experience worse pain that, unfortunately, often ends up ignored in sexual pain–related platforms [28]. Designing to include these sexual perspectives is critical to foster inclusivity and may eventually help address some of the myths and misconceptions that often fuel stigma related to nonheterosexual forms of sexual encounters. On the contrary, user-interface design practices that exclude such groups from web platforms could be considered a form of marginalization through design [29]—a practice that may not only places people with nonheterosexual experiences into isolation but also amplifies the stigma associated with such sexual encounters.

The use of nonprovocative images was seen as a good design strategy for addressing stigma. We understand that intervention developers may want to display anatomical images of sexual organs on web platforms for enhanced comprehension and clarity of the information presented [30]. While useful, such practices may need to be reconsidered in sexual health web platforms as they may inadvertently reproduce, perpetuate, or exacerbate stigmatized feelings among people who find such explicit content undesirable. Participants’ suggestion for the display of nonprovocative images supports our previous assertion that provocative and explicit sexual images on web platforms could inadvertently foment stigma among end users [7,15,22]. It further confirms the assertion that the stigma of sexual health issues may be amplified through digital content, particularly digital images [31]. Furthermore, even though there was a general desire for inclusive images of nonheterosexual encounters and gender-diverse people, care should be taken to ensure that such images are not provocatively displayed on digital platforms that may end up stigmatizing an already stigmatized group. A possible solution may involve user testing of such content among the different user populations before they are displayed on web platforms.

Participants did not only recommend the use of nonprovocative images but also expressed a desire for the colors of such images, and the general color schemes of web platforms, to be calming and nondistressing to users. The differences in color preferences might be a reflection of the diverse cultural backgrounds of the participants. Participants’ position on the use of calming colors like green, yellow, and blue confirms Goethe’s theory of colors where he associated certain color categories (eg, yellow and green) with positive emotional responses of warmth and excitement, while other categories (eg, red) were associated with negative arousal [32,33]. Even though participants were not explicit on how the choice of colors can address stigma, the current theories on colors suggest that less distressful color schemes like green and yellow could have a healing effect on people who have experienced the trauma of painful sex [33]. While we identified what is considered as calming colors, the differences in these colors might also pose a new challenge in determining a unified color scheme that is considered calming to a significant majority of web users. Thus, further research is needed to examine how the visual effects and different combinations of colors on user interfaces could alleviate the stigma of sexual pain.

Interpersonal communication features have been shown to provide informational support, instrumental support, belonging support, self-esteem, and emotional support to people with similar health conditions [34]. Participants’ recommendation for the integration of anonymous and interpersonal communication features (chat rooms, forum posts, and messaging boards) confirms the use of these interventions for people with other stigmatized conditions like HIV and AIDS [35]. The recommendations for networking functions in web platforms also support the use of social networking sites as an effective educational strategy for reaching out to people with diverse sexual experiences who often experience stigma [35]. With the increasing use of digital health technologies, such interpersonal connection features might move a significant part of sexual health services onto web-based platforms (eg, consultation, testing, and prescription). Beyond that, such interaction features could also foster new friendships for isolated individuals while providing opportunities for people to share and validate their personal experiences that are often internalized as negative feelings. These opportunities for interpersonal connection might not only make people feel less isolated but may also normalize their sexual pain experiences, thereby alleviating stigma [36].

Forums could have more effect on stigma reduction when people with internalized negative feelings can read personal accounts and testimonials from other people with the same disorder. Such testimonials may not just be limited to forums or messaging boards but could be displayed on landing pages, particularly if they are coming from popular personalities. Text or video testimonials from “famous” personalities have been shown to be very persuasive and might help elicit healthy emotions and memory [37]. Testimonials from famous personalities are especially important given the significant role of power and power dynamics in stigma formation [38]. Web-based video testimonials have shown effectiveness in alleviating explicit and implied stigma of mental illness among students [39], thus suggesting their usefulness for other stigmatized disorders in sexual health. Despite the importance of interpersonal connections on web platforms, it is important to be aware of the inherent security and privacy risks that could inadvertently expose people to further stigma or restigmatization [40]. The use of anonymous chat rooms and forum posts and moderation of web-based platforms by trained personnel is recommended to prevent the privacy risk associated with such platforms [41].

### Strengths and Limitations

Our study relied on web-based data collection methods, which provided some degree of anonymity and might have boosted the confidence of participants to discuss a sensitive topic like painful sex. We believe that the diversity of our sample may have enriched our findings by bringing a nuanced perspective of gender-diverse and nonheterosexual experiences of painful sex and their associated stigmatized feelings. Males engaging in nonheterosexual sexual acts like anal sex or females using sex toys may experience worse forms of stigma and sexual pain, yet are often ignored on web platforms.

Despite these strengths, this study was not without limitations. Even though we included participants who identified as men, two-spirit, and nonheterosexual people, their involvement was limited to 2 and 3 participants, respectively. The relatively lower participation rate could not have allowed us to delve deeper into the unique perspective of two-spirit and people who identify as men on how to address stigma via web-based platforms. Thus, further studies are needed to examine sexual pain stigma specific to each minority group (men, two-spirit, and nonheterosexual people) and highlight ways to incorporate the findings into web-based platforms. By just showing participants only our sample website, we also believed their responses might have been limited to a single website. It is possible that participants’ responses might have differed if they had been shown other websites on sexual pain. Despite this limitation, using a single website helped participants, particularly those who might not have used sexual pain–related websites before to be more articulate on how to develop websites to alleviate stigma.

### Conclusions

Web-based platforms on sexual pain have the potential to empower people, promote the development of new web-based friendships, and promote the sharing of personal experiences, thus reducing feelings of isolation. However, the manner in which these platforms are developed has implications by either alleviating or exacerbating the stigma of sexual pain. This study highlighted patient-centered design approaches that could serve as a reference guide in developing destigmatizing web platforms for people with sexual pain, particularly for those who feel stigmatized by their gender, racial, and sexual identity. While this study was conducted in the context of sexual pain, we believe that the results may also be applicable to web platforms on other stigmatized sexual health–related disorders or conditions and to stigmatized issues in mental health. As a next step, intervention studies may be needed to investigate how these guidelines might be applied in practice and whether web platforms resulting from these findings would indeed be less stigmatizing compared to websites that did not adopt such destigmatizing approaches.

## Appendix

Multimedia Appendix 1 Interview guide.
