# Supplementary material for: Patient-Centered Approaches for Designing Destigmatizing Sexual Pain-Related Web-Based Platforms: Qualitative Study
Source: JMIR Form Res. 2024 Mar 15;8:e53742. doi: 10.2196/53742 (PMC10980992; doi:10.2196/53742)
Supplement: Multimedia Appendix 1 [file formative_v8i1e53742_app1.docx]

**Interview guide**

Thank you for your interest in our study which seeks to understand your opinions on how websites on sexual pain can be designed to prevent or alleviate stigma. This interview will last approximately 45-60 minutes. We will be asking you questions related to how websites can alleviate stigma. Your responses are very valuable in developing the website to suit the needs of other users

1. What are your experiences with the use of sexual pain related websites or any other health-related website.
2. What role can digital technologies play in alleviating the stigma of sexual pain
3. What are your thoughts regarding how the information on digital platforms can be presented to prevent or alleviate stigma.
4. How can the interface appearance of sexual pain websites like colours, images, and text help in addressing preventing stigma?
5. Are there ways in which gender, sex and class representation on digital platforms can help in alleviating stigma?
6. How can the interactive features on web-platforms be used to alleviate stigma?
7. Do you have other suggestions on how websites can be developed to reduce stigma
